# Low- and Very-Low-Calorie Diets and Medication Use in Hospitalized Patients with Obesity: A Cross-Sectional Study

**DOI:** 10.3390/healthcare13111336

**Published:** 2025-06-04

**Authors:** Sérgio de Queiroz Braga, Márcia Cristina Almeida Magalhães Oliveira, Matheus Jorgetti Chamorro, Najara Araújo de Jesus, Rodrigo Almeida Magalhães Oliveira, Dandara Almeida Reis da Silva, Domingos Lázaro Souza Rios, Magno Merces

**Affiliations:** 1 Hospital da Obesidade, Camaçari 42825-901, Brazil; sergioqb@gmail.com (S.d.Q.B.); mcamoliveira@uneb.br (M.C.A.M.O.); ojuara.arajan@gmail.com (N.A.d.J.); darsilva@uneb.br (D.A.R.d.S.); 2 Departamento de Ciência da Vida, Universidade do Estado da Bahia (UNEB), Salvador 41720-040, Brazil; matheus-jorgetti@hotmail.com (M.J.C.);; 3 Programa de Pós-Graduação em Ciências da Saúde, Faculdade de Medicina da Bahia, Universidade Federal da Bahia (UFBA), Salvador 40110-909, Brazil; rodrigoalmaoli@gmail.com

**Keywords:** severe obesity, ketogenic diet, very-low-calorie diet, hospitalization, polypharmacy, lifestyle intervention

## Abstract

**Background**: Obesity is a growing global health concern associated with numerous comorbidities and high medication burden. This study aimed to evaluate the impact of low- and very-low-calorie diets (LCD/VLCD), combined with intensive lifestyle changes, on comorbidities and medication use in hospitalized patients with class II and III obesity. **Methods**: A retrospective cohort study was conducted using medical records of patients hospitalized for 3–6 months at a specialized obesity hospital in Brazil. Prescription data for antihypertensive, hypoglycemic, and lipid-lowering drugs were compared at admission, 3, and 6 months. Descriptive statistics, chi-squared tests, and *t*-tests were used to compare medication use and weight change over time. **Results**: Among 246 patients, the proportion of those using antihypertensives decreased from 74.4% at admission to 44.7% at 6 months (*p* < 0.02), with significant reductions also observed at 3 months (*p* < 0.001). Hypoglycemic prescriptions also declined at 3 months (*p* = 0.01), but not significantly at 6 months. Lipid-lowering medication use showed no significant changes. Average weight loss was 11% at 3 months and 21.3% at 6 months. **Conclusions**: Hospitalization with LCD/VLCD and lifestyle therapy was associated with a short-term reduction in medication burden, especially antihypertensives, supporting the potential of inpatient multidisciplinary strategies for severe obesity management.

## 1. Introduction

Obesity is defined by the World Health Organization (WHO) as a chronic and multifactorial disease, with a body mass index (BMI) ≥ 30 kg/m^2^ [1]. As BMI increases, so do morbidity and mortality risks, with values ≥35 kg/m^2^ and ≥40 kg/m^2^ corresponding to class II and class III obesity, respectively [2,3], as defined for adult populations aged 18 years and older. This is a condition of growing epidemiological and social relevance, affecting over 890 million adults in 2022 [1]. The number of adults affected by obesity has nearly tripled since 1975, indicating a sustained global upward trend. In 2019, obesity was associated with approximately 5 million deaths from non-communicable diseases [4], highlighting its magnitude as a global public health problem.

The main comorbidities associated with obesity include cardiovascular diseases, type 2 diabetes mellitus, insulin resistance, dyslipidemia, respiratory disorders, and osteoarticular, gastroesophageal, and neurological conditions, in addition to certain types of cancer and psychiatric disorders [1,3,5]. The economic impact is also significant: the costs of primary and specialized care place a heavy burden on health systems. In Brazil, between 2008 and 2010, annual expenditures on obesity-related medical care exceeded USD 1.1 billion [5,6,7]. Although these data are not recent, they remain among the few national estimates available for the economic impact of obesity in Brazil.

Lifestyle modification is considered the gold-standard treatment for obesity, involving dietary, behavioral, and regular physical activity strategies [8,9]. In more advanced cases, pharmacotherapy and bariatric surgery are recommended as adjunct therapies [10,11]. In this context, hospitalization with a multidisciplinary approach has shown efficacy in the management of class II obesity [9,10,11,12]. Among dietary interventions, low-calorie diets (LCDs) and very-low-calorie diets (VLCDs) stand out, promoting severe carbohydrate restriction (<50 g/day), protein intake proportional to ideal body weight (1–1.5 g/kg), reduced fat intake (15–30 g/day), and an energy intake between 500 and 800 kcal/day [8,12]. This strategy induces ketosis and has been associated with greater weight loss and comorbidity reduction, surpassing the results of traditional hypocaloric diets in randomized clinical trials [13].

Although LCDs and VLCDs differ in energy intake thresholds, both strategies were managed using the same multidisciplinary inpatient protocol in our institution, with shared nutritional goals, clinical supervision, and behavioral support. For this reason, LCDs and VLCDs were grouped as a single intervention in our analyses.

In addition to their well-documented impact on weight loss and glycemic control, recent studies have also demonstrated a reduction in pharmacological burden associated with LCDs and VLCDs. In a 12-week clinical trial, Gomez-Arbelaez et al. (2017) [14] observed that patients consuming a VLCD significantly reduced or discontinued antihypertensive and hypoglycemic agents following marked metabolic improvement. Similar findings were reported by Lorenzo et al. (2025) [15], who documented medication de-escalation among patients following a ketogenic VLCD protocol over two years of follow-up.

This study aimed to evaluate the impact of low- and very-low-calorie diets (LCDs/VLCDs), combined with intensive lifestyle changes, on the use of medications for cardiometabolic comorbidities in patients with class II and III obesity hospitalized for at least three months at the Hospital da Obesidade in Camaçari, Bahia, Brazil.

Structured inpatient treatment programs for class II and III obesity using LCD and VLCD protocols have been in place at the Hospital da Obesidade since 2016. Patients typically remain hospitalized for three to six months, depending on clinical response and administrative criteria. This model provides a controlled setting for intensive lifestyle intervention.

## 2. Materials and Methods

This is a retrospective cohort study with a pre–post intervention design, conducted from secondary data in electronic medical records of patients hospitalized between October 2016 and October 2022 for the treatment of obesity using low-calorie (LCDs) and very-low-calorie diets (VLCDs). The study was conducted at the Hospital da Obesidade, a specialized institution for the clinical management of severe obesity and related comorbidities, offering full-time hospitalization and individualized multidisciplinary care.

The study used a convenience sample composed of individuals with obesity who remained hospitalized for three to six months. Inclusion criteria were age over 12 years; diagnosis of class II obesity (BMI between 35 and 39.9 kg/m^2^) or class III obesity (BMI ≥ 40 kg/m^2^) at admission; treatment with LCDs or VLCDs, either continuously or intermittently; use of hypoglycemic, antihypertensive, and/or lipid-lowering drugs at admission; and bioimpedance analysis performed at admission, and at the third and/or sixth month of hospitalization. Patients with incomplete data in their medical records or a length of stay under three months were excluded. No specific exclusion criteria were applied based on the severity of comorbidities. However, no patients included in this study presented with decompensated psychiatric conditions, active cancer, advanced chronic kidney disease, or heart failure. All participants met the institutional clinical criteria for hospitalization and were considered eligible for dietary and lifestyle interventions.

Only patients who remained hospitalized for at least three or six full months were included in the respective analyses. Thus, the assessments at three and six months refer to standardized timepoints during the inpatient treatment protocol. These do not represent variable periods following hospital discharge. This methodological approach ensures comparability across patients at each evaluation stage.

The primary outcomes were changes in the prescription of antihypertensive, hypoglycemic, and lipid-lowering drugs between admission and the third and/or sixth month of hospitalization. These changes included both complete discontinuation and the reduction in the number of medications prescribed per patient, regardless of dosage adjustments. The total number of pharmacological agents used by each individual was counted at each timepoint and classified by drug class.

Treatment was carried out by a multidisciplinary team and included LCD and VLCD protocols, both with increased protein intake (70 to 100 g/day or 0.8 to 1.5 g of protein per kg of ideal body weight per day), low carbohydrate content, and supplementation with vitamins, minerals, electrolytes, and essential fatty acids, according to national guidelines. The intervention also included supervised physical activity (water aerobics, recumbent cycling, and resistance training), psychological support with Cognitive Behavioral Therapy (twice a week), daily educational group meetings, and continuous multiprofessional clinical evaluation, with follow-up by specialist physicians, physiotherapists, occupational therapists, nutritionists, and nursing staff.

Data collection was based on medical prescriptions recorded at admission and following three months and six months of hospitalization. Data were extracted from electronic records, organized in Microsoft Excel spreadsheets, and exported to SPSS version 29 for Windows. Medications were classified by active ingredient and dosage. Pharmacological classification was performed manually by grouping medications into standard therapeutic classes based on their mechanism of action, as defined in national formularies and clinical guidelines.

After hospital discharge, patients were referred to a structured outpatient follow-up program at the same institution. Nutritional counseling, psychological support, and clinical monitoring were provided monthly, through both teleconsultations and in-person evaluations, for a period of up to two years. This post-discharge support is intended to reinforce adherence to the dietary and behavioral modifications initiated during hospitalization.

The evaluation timepoints at three and six months were chosen based on the standardized inpatient treatment protocol used at the institution. The three-month mark represents the minimum length of stay required to complete the intensive dietary and behavioral intervention. The six-month point corresponds to the maximum hospitalization period typically approved by the clinical care plan and health insurance providers. These intervals allow for an assessment of both short-term and extended responses to the intervention.

Descriptive analysis of pharmacological class frequencies was performed at each evaluation point (admission, three, and six months). Additionally, an individual analysis of medication count per patient over time was conducted. Comparisons between periods were performed using Fisher’s exact test, with or without Monte Carlo simulation, when indicated. *p*-values were calculated for comparisons between admission and three months, admission and six months, and between three and six months, as presented in the results.

This research followed the ethical principles established by Resolution No. 466/2012 of the Brazilian National Health Council (CONEP/Brazil) and the precepts of the Declaration of Helsinki. The project was approved by the Ethics Committee of the State University of Bahia (UNEB), under opinion number 7.498.693.

## 3. Results

From an initial population of 1151 hospitalized patients, 246 individuals with class II or III obesity were included after applying inclusion and exclusion criteria. Among them, 75.6% were female. The mean age was 43.2 ± 11.7 years, ranging from 13 to 72 years. All patients remained hospitalized for at least three months and up to six months, depending on clinical progression and treatment planning. Most participants had a history of multiple comorbidities.

Thirty-four different substances were identified among antihypertensive drugs, distributed across seven pharmacological classes; fourteen hypoglycemic agents from six distinct classes; and eight lipid-lowering drugs distributed across three classes (as shown in Table 1, Table 2 and Table 3, respectively). The average weight loss at three months was approximately 11% of initial body weight, and at six months, 21.3%. Although the sample included patients with both class II and class III obesity, weight loss was analyzed as an average across the entire cohort. Stratified analyses by obesity class were not performed, as the primary objective was to evaluate medication changes in patients undergoing a standardized inpatient protocol. Future studies may explore whether the magnitude of weight loss differs by obesity severity.

The class of angiotensin II receptor blockers (ARBs) was the most frequently used throughout the entire evaluation period, with proportions ranging from 34.5% at admission to 43% at three months, and remaining at 37.5% at six months. Diuretics and calcium channel blockers were the second and third most frequently prescribed classes, respectively. Neprilysin inhibitors were used only at admission and were subsequently discontinued (Table 1).

A significant reduction in the number of antihypertensives used per patient was observed during hospitalization. One patient who used six medications at admission reduced to just one by the sixth month. In general, most patients reduced by at least one antihypertensive medication between admission and the third month, and this reduction was maintained or increased by the sixth month. The proportion of patients not using any antihypertensives increased from 25.6% at admission to 55.3% at six months. Statistical analyses showed significant reductions in antihypertensive use between admission and three months (*p* < 0.001), admission and six months (*p* < 0.02), and between the third and sixth months (*p* < 0.02).

At admission, biguanides were the most prescribed hypoglycemics (59%), followed by SGLT2 inhibitors (22.2%) and GLP-1 receptor agonists (7.6%). At three months, GLP-1 agonists became predominant (57.5%), surpassing biguanides (15.9%), while the use of SGLT2 inhibitors remained stable. At six months, nearly 70% of patients were using GLP-1 agonists, while the use of biguanides and SGLT2 inhibitors dropped to 11.6% each (Table 2).

There was a significant reduction in the number of hypoglycemic agents used between admission and the third month (*p* = 0.01). Of the five patients using three hypoglycemic drugs at admission, four reduced to one or none. Among those using two, 96.3% reduced their medication count after three months. However, this trend did not remain statistically significant at six months (*p* = 0.2), nor in the comparison between the third and sixth months (*p* = 0.08).

Statins were the predominant class among lipid-lowering drugs throughout all evaluated periods, representing 80.5% at admission and 73.3% at six months. Fibrates were the second most used class at admission, while cholesterol absorption inhibitors (ezetimibe) ranked second at three and six months (Table 3).

Despite the trend toward a reduction in the number of lipid-lowering medications used, the differences observed between periods were not statistically significant. Most patients who used these drugs at admission discontinued them by the third and sixth months, but Fisher’s exact test showed no significant differences in comparisons (*p* = 0.33; *p* = 0.38; *p* = 0.08, respectively).

## 4. Discussion

This study aimed to assess the impact of hospital-based intervention using low- and very-low-calorie diets, combined with intensive lifestyle modification, on medication use for comorbidities in patients with class II and III obesity. The findings indicate a significant reduction in the use of antihypertensive drugs, and to a lesser extent, hypoglycemic agents, particularly during the first three months of treatment.

Among antihypertensives, ARBs were the most prevalent class at both admission and after three months, consistent with Brazilian Hypertension Guidelines, which recommend this class as a first-line option, particularly for patients with comorbidities, due to its safety profile and nephroprotective effects [16]. Thiazide diuretics and calcium channel blockers (CCBs) followed as the most prescribed classes, also in line with national recommendations and evidence such as the ALLHAT study [17,18]. The limited use of neprilysin inhibitors is likely due to their restricted indication for heart failure with reduced ejection fraction [16,19].

After three and six months of hospitalization, a significant reduction in the number of antihypertensive medications was observed, even among patients using up to six drugs at admission. The average weight loss at three and six months was 11% and 21.3%, respectively, supporting findings from meta-analyses that demonstrate blood pressure reductions with modest weight loss [20]. Thirty-three patients who initially used only one medication were able to discontinue it, possibly due to the resolution of borderline hypertension. On the other hand, 14 patients who did not use antihypertensives at admission started treatment, potentially due to delayed adherence, improved access to medical care, or increased awareness of their condition following hospitalization [10,12,16,18,21,22].

Regarding hypoglycemics, biguanides were the most commonly prescribed at admission, followed by SGLT-2 inhibitors and GLP-1 receptor agonists, in accordance with ADA guidelines for T2DM management [20]. From the third month, a shift in prescription patterns was observed, with a notable increase in the use of GLP-1 receptor agonists, whose efficacy for glycemic control and weight loss is well documented [23,24]. The reduction in biguanide use and the increased prescription of weight-loss-promoting agents (such as GLP-1) reflect a therapeutic redirection after glycemic stabilization.

Insulins and DPP-4 inhibitors were infrequently used, likely due to the clinical profile of patients and the emphasis on therapies promoting weight loss. The substitution by GLP-1 receptor agonists is justified not only by efficacy but also by additional metabolic benefits [25]. By the end of the third month, 33 patients had discontinued hypoglycemic medications. The lower discontinuation rate compared to antihypertensives may reflect the need for stricter glycemic control in patients with insulin resistance [26,27,28]. Additionally, 42 patients who were not on hypoglycemics at admission began therapy, possibly for weight loss purposes [24].

However, by six months, there was no statistically significant reduction in hypoglycemic use. This plateau may indicate that most treatment response occurs in the early months, requiring additional strategies to sustain or enhance clinical effects over time [12,29].

As for lipid-lowering agents, statins remained the most prescribed class throughout the six months, as expected given their first-line role in lowering LDL in high-risk cardiovascular patients, such as those with obesity [30]. Continued statin prescription after weight loss may relate to the presence of comorbidities (e.g., hepatic steatosis, diabetes, hypertension), which maintain elevated residual risk [16,30,31]. Therefore, despite clinical improvement and reduced use of other medications, statins remain necessary for most patients.

This study’s limitations include the absence of a control group, which limits direct causal attribution between interventions and outcomes. The retrospective design may also be subject to selection and recording bias. Additionally, the use of a convenience sample may have introduced selection bias and limits the generalizability of the findings to broader populations with obesity. Patients included in the study were those who completed at least three months of hospitalization, which may represent a subgroup with higher adherence, clinical stability, or specific insurance coverage. Furthermore, the data collected at three and six months correspond to fixed evaluation milestones during hospitalization, not post-discharge intervals. This design choice reduces the variability that might arise from uneven follow-up timing but limits our ability to assess long-term maintenance of treatment effects after discharge. Prospective, controlled studies are recommended to better elucidate the impact of hypocaloric diets and intensive hospitalization on reducing the pharmacological burden in patients with severe obesity.

From a practical perspective, the findings reinforce the role of hospitalization with an intensive multidisciplinary approach as a viable and effective clinical strategy for patients with class II and III obesity, particularly in reducing medication use for cardiometabolic comorbidities. The significant reduction in antihypertensive use and restructuring of hypoglycemic therapy observed within the first three months suggest that structured interventions with LCD and VLCD, physical activity, and behavioral support may promote not only weight loss but also improved chronic disease control. Clinically, these data contribute to more personalized therapeutic decisions, enabling safe deprescription in a monitored environment, reducing costs, improving adherence, and decreasing the risks associated with polypharmacy.

## 5. Conclusions

The treatment of obesity through hospitalization with low- and very-low-calorie diets, combined with lifestyle modification, reduced the use of antihypertensives and hypoglycemic agents during the first three months of treatment in patients with class II and III obesity. There was no significant reduction in the use of lipid-lowering drugs. The findings suggest short-term clinical benefits but highlight the need for prospective, controlled studies with longer follow-up.

## Figures and Tables

**Table 1:** Use of antihypertensive drugs at admission, 3 and 6 months—Hospital da Obesidade, Camaçari, Brazil (2016–2022).

Class	Admission (%)	3 Months (%)	6 Months (%)
ARBs	34.5	43.0	37.5
Beta-blockers	13.8	9.9	14.1
Calcium channel blockers	17.1	18.3	20.3
Diuretics	28.1	21.8	23.4
ACE inhibitors	2.6	4.2	3.1
Neprilysin inhibitors	0.5	-	-
Vasodilators	3.4	2.8	1.6
Statistical comparison	Admission vs. 3 months (*p* < 0.001); admission vs. 6 months (*p* < 0.02); 3 vs. 6 months (*p* < 0.02)		

Values represent the percentage of patients using each drug class at each timepoint (n = 246).

**Table 2:** Use of hypoglycemic agents at admission, 3, and 6 months—Hospital da Obesidade, Camaçari, Brazil (2016–2022).

Class	Admission (%)	3 Months (%)	6 Months (%)
Biguanides	59.0	15.9	11.6
GLP-1 Agonists	7.6	57.5	69.8
Insulin	2.1	0.9	4.7
SGLT2 Inhibitors	22.2	22.1	11.6
Sulfonylureas	4.2	-	-
DPP-4 Inhibitors	4.9	3.5	2.3
Statistical comparison	Admission vs. 3 months (*p* = 0.01); admission vs. 6 months (*p* = 0.2); 3 vs. 6 months (*p* = 0.08)		

Values represent the percentage of patients using each drug class at each timepoint (n = 246).

**Table 3:** Use of lipid-lowering drugs at admission, 3, and 6 months—Hospital da Obesidade, Camaçari, Brazil (2016–2022).

Class	Admission (%)	3 Months (%)	6 Months (%)
Cholesterol absorption inhibitors	8.8	18.2	20.0
Fibrates	10.6	4.5	6.7
Statins	80.5	77.3	73.3
Statistical comparison	Admission vs. 3 months (*p* = 0.33); admission vs. 6 months (*p* = 0.38); 3 vs. 6 months (*p* = 0.08)		

Values represent the percentage of patients using each drug class at each timepoint (n = 246).

## Data Availability

Data are contained within the article.
